# The Wnt pathway regulator DKK1 is preferentially expressed in hormone-resistant breast tumours and in some common cancer types

**DOI:** 10.1038/sj.bjc.6603579

**Published:** 2007-01-23

**Authors:** M-A Forget, S Turcotte, D Beauseigle, J Godin-Ethier, S Pelletier, J Martin, S Tanguay, R Lapointe

**Affiliations:** 1 Research Centre, Centre hospitalier de l'Université de Montréal (CHUM) – Hôpital Notre-Dame, Department of Medicine, Université de Montréal, and Institut du cancer de Montréal, Montréal, Québec, Canada; 2 McGill University Health Centre, Montreal General Hospital, Montréal, Québec, Canada

**Keywords:** Dickkopf-1 (DKK1), breast cancer, lung cancer, kidney cancer, prognostic and diagnostic marker

## Abstract

In addition to new tumour antigens, new prognostic and diagnostic markers are needed for common cancers. In this study, we report the expression of Dickkopf-1 (DKK1) in multiple common cancers. This constitutes a comprehensive analysis of the DKK1 expression profile. Dickkopf-1 expression was evaluated by classical and quantitative reverse transcriptase–polymerase chain reaction (RT–PCR) and enzyme-linked immunosorbant assay for protein determination, in cancer lines and clinical specimens of several cancer origins. For breast cancer, expression was correlated with clinicopathological parameters. Dickkopf-1 expression was confirmed in several cancer cell lines derived from breast and other common cancers. Dickkopf-1 protein secretion was documented in breast, prostate and lung cancer lines, but was negligible in melanoma. Analysis of DKK1 expression in human cancer specimens revealed DKK1 expression in breast (21 out of 73), lung (11 out of 23) and kidney cancers (six out of 20). Interestingly, DKK1 was preferentially expressed in oestrogen and progesterone receptor-negative tumours (ER^−^/PR^−^; *P*=0.005) and in tumours from women with a family history of breast cancer (*P*=0.024). Importantly, DKK1 protein production was confirmed in multiple breast cancer specimens that were positive by RT–PCR. This work establishes DKK1 as a potential prognostic and diagnostic marker for cohorts of breast cancer patients with poor prognosis. Dickkopf-1 may also become a relevant candidate target for immunotherapy of different cancers.

Neoplasia frequently results in an aberrant protein expression profile, including proteins involved in embryogenesis. For example, *α*-fetoprotein, which is a fetal serum protein (Taketa, 1990), is also expressed in hepatocellular carcinoma. Frequently, such genes involved in embryogenesis and fetal development are re-activated in tumours and may be implicated in the neoplasia process. The expression of some of these proteins can be exploited as tumour markers (Gorog *et al*, 2005), serves in diagnosis, prognosis and in monitoring of relapse or treatment effectiveness. Specifically, very few secreted tumour markers are available for the management of common cancers. In addition, such highly specific secreted proteins could be targeted as tumour antigens (TA) for tumour immunotherapy.

Here, we report the expression of Dickkopf-1 (DKK1) in breast cancer and other tumours. Dickkopf-1 is a secreted protein involved in embryonic development. Specifically, Wnt-1 protein binds to the frizzled receptor (Fz) and the low-density lipoprotein receptor-related protein-5/6 (LRP5/6), triggering signals important for proliferation via *β*-catenin. Dickkopf-1 binds to LRP5/6 (Semenov *et al*, 2001) and blocks interaction with Wnt-1 resulting in *β*-catenin degradation and effects on proliferation (Mao *et al*, 2002). Interestingly, DKK1 expression in cancer has been described previously, mainly in multiple myeloma (Tian *et al*, 2003), hepatoblastomas and Wilms' tumours (Wirths *et al*, 2003).

In the present work, we exploited publicly available expression tissue libraries with digital differential display bioinformatic tools to highlight genes specifically expressed in breast cancer but absent from normal tissues critical for body functions. Among the different genes listed, we confirmed that DKK1 was expressed in breast cancer cells, with restricted expression in the placenta. Dickkopf-1 appears to be preferentially expressed in hormone-independent tumours and in tumours from women with a family history of breast cancer. Interestingly, the expression of this gene has been confirmed in cancers of other origins, such as the lung, kidney and melanoma. This work establishes DKK1 as a potential prognostic and diagnostic marker of aggressive breast cancer types. In addition, DKK1 could be valuable for detecting lung and kidney cancers, for which no reliable secreted marker is available. Finally, DKK1 has become a relevant candidate target for immunotherapeutic approaches to different cancers, and it may also have potential in a preventive vaccination strategy for women at high risk of developing breast cancer.

## MATERIALS AND METHODS

### Bioinformatic tools for differential gene expression in breast cancer compared to normal tissues

To find candidate genes, we exploited the Digital Gene Expression Displayer (DGED), a bioinformatic tool freely available from the Cancer Genome Anatomy Project server (http://www.ncbi.nlm.nih.gov/ncicgap/) (Strausberg *et al*, 2000), probing two different complementary DNA (cDNA) expression libraries, expressed sequence tag (EST; http://cgap.nci.nih.gov/Tissues/GXS) and serial analysis of gene expression (SAGE; http://cgap.nci.nih.gov/SAGE/SDGED?METHOD=SS10,LS10&ORG=Hs). These bioinformatic tools allowed the analysis of expression profiles from the EST and SAGE databases by the clustering of libraries by origin. All the available libraries prepared from normal tissues were clustered in one group, and all available libraries prepared from breast cancers were clustered in a second distinct group. Candidate genes were selected on the basis of high expression levels in available human breast cancer libraries, and absent or low levels in normal human tissues from important organs.

### Cell culture

The breast cancer cell lines MCF-7, MDA231, BT-20, HCC1428 BRCA and HCC2218 BRCA were obtained from the American Type Culture Collection (ATCC, Manassas, VA, USA). B lymphocytes immortalised with Epstein–Barr virus (EBV) HCC1428 and HCC2218, as well as the lung cancer lines Calu6, H1299, A549, H460 and H596, the prostate cancer lines DU145, PC-3 and LNCaP, and the human embryonic kidney (HEK) line 293T were obtained from the ATCC. The melanoma lines 397mel, 537mel, 586mel, 888mel, 1087mel, 1088mel, 1278mel, 1300mel, 1337mel and MelS-FB and the kidney cancer line RCC-W were all established at the Surgery Branch of the National Cancer Institute/National institute of health, and the SK23 line was acquired from the ATCC. The ovarian cancer line SKOV3 was also kindly provided by the Surgery Branch. Most of the cell lines were cultured in RPMI 1640 (Invitrogen; Carlsbad, CA, USA; and Wisent, St-Bruno, Québec, Canada) supplemented with 10% heat-inactivated foetal bovine serum (Invitrogen and Wisent), 2 mM L-glutamine, 100 U ml^−1^ penicillin/streptomycin (Invitrogen) and 10 *μ*g ml^−1^ gentamicin (Invitrogen). For the HCC breast cancer lines and their corresponding EBV-B lines, 10 mM HEPES solution (Invitrogen) and 1 mM sodium pyruvate (Invitrogen) were added to the culture medium.

Peripheral blood mononuclear cells (PBMC) were collected from healthy donors recruited by Dr Jean-Pierre Routy at the McGill University Health Centre (MUHC, Royal Victoria Hospital, Montréal, Québec, Canada). The PBMC were prepared from blood by centrifugation on a lymphocyte separation medium (Cellgro, Herndon, VA and Wisent). To generate CD40-activated B cell cultures (CD40-B), B cells from bulk PBMC were cultured with 500 ng ml^−1^ soluble trimeric CD40L (Immunex Corporation, Seattle, WA, USA) and 250 U ml^−1^ recombinant human IL-4 (Peprotech, Rocky Hill, NJ, USA) in Iscove's modified Dulbecco's medium (Invitrogen) supplemented with 7.5% human serum (heat-inactivated, prepared from normal donors), 2 mM L-glutamine, 100 U ml^−1^ penicillin/streptomycin and 10 *μ*g ml^−1^ gentamicin. Fresh Iscove medium was added on day 3 with 250 U ml^−1^ IL-4 and 250 ng ml^−1^ CD40L as described previously (Lapointe *et al*, 2003). For T lymphocyte cultures, PBMC were incubated in complete medium consisting of AIM-V medium (Invitrogen) supplemented with 5% human AB serum (heat-inactivated; Gemini Bio-Products, Calabasas, CA, USA), 2 mM L-glutamine, 100 U ml^−1^ penicillin/streptomycin and 10 *μ*g ml^−1^ gentamicin (all from Invitrogen), and supplemented with 300 IU ml^−1^ recombinant human IL-2 (Chiron, Emeryville, CA, USA) and 30 ng ml^−1^ of agonistic anti-CD3 (OKT3, eBiosciences, San Diego, CA, USA) or 5 *μ*g ml^−1^ of phytohemagglutinin (PHA; Sigma, Oakville Ontario, Canada).

### Clinical specimens

Breast cancer specimens of 1.5 cm or higher were provided by the Fonds de la recherche en santé du Québec (FRSQ) Cancer Network breast tissue library of CHUM – Hôpital Notre-Dame and Hôpital Hôtel-Dieu (specimens stabilised in RNAlater™, Sigma; for reverse transcriptase–polymerase chain reaction (RT–PCR) analyses). Lung cancer specimens were obtained after resection in the Thoracic Surgery Department of CHUM – Hôpital Notre-Dame (five samples; stabilised in RNAlater), and all others were acquired from the Lung Cancer Tissue Library of the FRSQ Respiratory Health Network of Hôpital Laval (Québec, Québec, Canada; snap-frozen tumour pieces). Kidney cancer specimens were collected after partial or total kidney resection at the Montreal General Hospital (MUHC).

Snap-frozen and RNAlater-stabilised cancer samples were homogenised with a Medimachine™ (Dako Cytomation, Glostrup, Denmark) according to the manufacturer's instructions. RNA was prepared with Qiazol reagent (QIAGEN GmbH, Hilden, Germany), followed by a cleanup and concentration procedure using the RNeasy™ Mini or Micro Kit (QIAGEN) according to the manufacturer's instructions.

The status of the oestrogen and progesterone receptors in the breast cancer specimens was determined by the clinical pathology services of CHUM – Hôpital Notre-Dame and Hôpital Hôtel-Dieu.

### Reverse transcriptase–polymerase chain reaction

RNA from cell lines and lymphocytes was prepared with Rneasy™ Mini or Micro Kits (QIAGEN) according to the manufacturer's instructions. Intron-spanning PCR primers were designed from genes selected by the bioinformatic approach. To perform classical and quantitative RT–PCR analyses, cDNA was first synthesised from mRNA (1 *μ*g) with oligo-dt (Invitrogen) using an Omniscript Reverse Transcriptase Kit (QIAGEN). Classical RT–PCR amplification was undertaken with the HotStartTaq DNA Polymerase (QIAGEN). The cycling conditions were 15 min at 95°C, 24 (*β*-actin) or 32 (DKK1 and other genes) cycles of 45 s at 94°C, 45 s at 55°C, 1 min at 72°C, with a final extension of 10 min at 72°C, in a T3 Thermocycler™ system (Biometra, Goettingen, Germany). The primer sequences for *β*-actin were: 5′: GGAAGGCTGGAAGAGTGCC; and 3′: GTGATGGTGGGCATGGGTC, resulting in a 700-bp amplicon. Amplification was detected by ethidium bromide staining after electrophoresis migration in agarose gel (1.5 or 2%; with apparatus from Bio-Rad, Hercules, CA, USA). The primer sequences were as follows: DKK1 (5′ primer: ATTCCAACGCTATCAAGAACC; 3′ primer: CCAAGGTGCTATGATCATTACC, amplicon 383 bp).

For quantitative real-time RT–PCR, amplification was performed in a LightCycler™ system (Roche, Mannheim, Germany) and revealed with an SYBR Green™ kit (Quantitect™ SYBR Green PCR, QIAGEN). Standard curves for each gene were established to quantify the number of copies for each sample, and expression was considered only when the sample *C*_t_ was within the limit of each standard curve. The cycling conditions were 15 min at 95°C, 40 cycles of 15 s at 94°C, 30 s at 55°C, 30 s at 72°C and 5 s at 82°C (*β*-actin) or 84°C (DKK1). The primer sequences for real-time PCR were *β*-actin 5′: AAGGCCAACCGCGAG; 3′: TAATGTCACGCACGATTCCCG; DKK1 5′: CTCGGTTCTCAATTCCAACG; 3′: GCACTCCTCGTCCTCTG. Finally, amplification of the relevant amplicon was confirmed by separation on agarose (2%) gel, revealed as mentioned earlier. *β*-actin was exploited as a housekeeping gene. A sample was considered positive for DKK1 when amplification was >200 copies of DKK1 per 10^5^ copies of *β*-actin. This threshold value was established considering that the expression levels in normal tissues were less than 200 copies, with the exception of the placenta.

### Enzyme-linked immunosorbent assay

Cancer cell lines and lymphocytes culture medium were tested for the presence of secreted DKK1 protein by enzyme-linked immunosorbent assay (ELISA). Cells were seeded at 1 × 10^5^cells well^−1^ in flat-bottom 96-well plates (Corning Inc., Corning, NY, USA). Supernatants were harvested after 24 h and assayed for DKK1 with the commercial DuoSet Human DKK1 ELISA Kit, as recommended by the manufacturer (R&D Systems, Minneapolis, MN, USA). The lowest standard point for the ELISA assay was 62 pg ml^−1^.

Dickkopf-1 protein production was also quantified in breast cancer lysates. Freshly resected breast cancer specimens provided by the FRSQ Cancer Network (as mentioned earlier) were mechanically homogenised in complete AIM-V medium with the Medimachine (Dako Cytomation) to obtain a single-cell suspension. Lysates were prepared by five rapid subsequent freeze/thaw cycles (Lapointe *et al*, 2003). Cell debris were sedimented, and supernatants were assayed for DKK1 as described above. Cell line lysates used as controls were prepared by the same technique, from 1 × 10^7^ cells ml^−1^ suspension.

### Statistical analyses

Mean values of DKK1 expression were compared by clinico-pathological clusters with the two-tailed *t*-test for independent samples. Dickkopf-1-positive tumours were compared to DKK1-negative tumours for the same clusters, using the two-sided Pearson χ^2^ test. Differences were considered significant at *P*<0.05. Statistics were performed with SPSS 13.0 software for Windows (LEAD Technologies, Chicago, IL, USA).

## RESULTS

### DKK1 expression in breast cancer cell lines

We originally intended to find new TA for potential applications in immunotherapy, by exploiting the DGED. This bioinformatic approach was used as a screening tool to highlight potential overexpressed genes in breast cancer. This list of genes predicted by these banks (Supplementary data; Supplementary Table 1) was considered as preliminary data needing to be further investigated. By using these mining tools, some genes predicted to be practically absent from normal tissues later showed expression in various normal tissues by RT–PCR analyses (e.g. Myl5 and S100A7 Supplementary data, Figure 1A and B). This demonstrates the importance of confirming the expression profile of genes predicted with the DGED.

**Figure 1:**
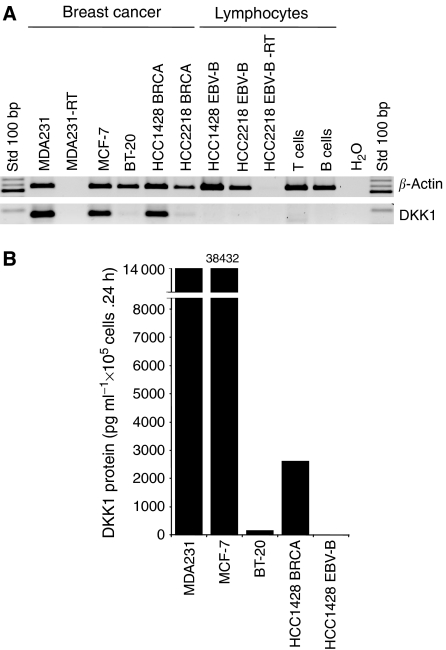
Expression profile of genes selected by the bioinformatic approach in tumour cell lines and PBMC. (**A**) Messenger RNA was prepared from the indicated cell lines, and RT–PCR analyses were performed with the specific primers indicated. Normal primary cell lines were prepared by stimulation of PBMC with anti-CD3 and IL-2 (T cells), or with soluble CD40L and IL-4, which stimulate B lymphocytes to proliferate (B cells). Reverse transcriptase was omitted in the MDA231-RT group and HCC2218 EBV-B cells (EBV-B−RT). HCC2218 EBV-B and HCC1428 EBV-B are EBV-immortalised B lymphocytes prepared from breast cancer patient HCC2218 and HCC1428, respectively. Amplification was detected by ethidium bromide staining after electrophoresis migration in agarose gel. The results presented are representative of at least three independent experiments. (**B**) Indicated tumour cell lines were plated for 24 h in 96-well plates as described in Materials and Methods. Supernatants were harvested, and DKK1 secretion was determined by ELISA. The results presented are an average of at least two independent experiments.

Dickkopf-1, a modulator of the Wnt pathway, emerged as a candidate overexpressed gene; however, to validate the SAGE database information, the expression profile was further analysed and confirmed by RT–PCR. RNA was first prepared from the different cell lines of breast cancer and other origins. Negative control cells included PBMC and cultured, activated lymphocytes to eliminate the possibility of this gene being expressed in proliferative cells. Intron-spanning PCR primers were designed for the RT–PCR analysis. Dickkopf-1 expression was confirmed in breast cancer lines but not in activated lymphocytes (Figure 1A). High DKK1 protein secretion was confirmed in culture supernatants harvested from three breast cell lines (MDA231, MCF-7 and HCC1428; Figure 1B). Interestingly, no DKK1 was detected in the HCC1428 EBV-B cell lines, and a very low amount was found in BT-20, which reflects the faint band in RT–PCR (Figure 1A).

In summary, we have validated the expression, in breast cancer lines, of a gene previously selected by the bio-informatic approach. Although TA are frequently expressed in both fresh tumour samples and tumour cell lines, confirmation of expression in fresh breast cancer specimens is essential. Also, limited expression in normal tissues needed to be evaluated.

### DKK1 is expressed in the placenta

**Figure 2:**
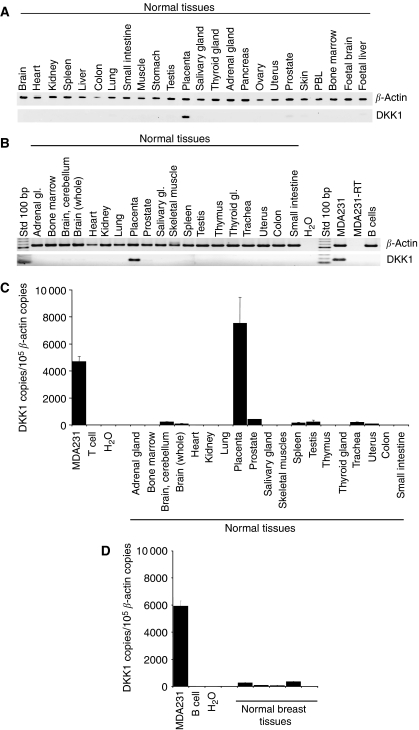
Expression profile of DKK1 in normal tissues. Messenger RNA was prepared from normal tissues (acquired from Origene in (**A**), and from BD-Clontech in (**B**–**D**) and controls. (**A** and **B**) Semiquantitative classical RT–PCR analyses were performed with *β*-actin and primers indicated as described in Materials and Methods. (**C**) Expression from normal tissues was evaluated by quantitative real-time RT–PCR. (**D**) Expression in normal breast tissues was also evaluated by quantitative real-time RT–PCR. The results presented are representative of at least two independent experiments.

Classical and quantitative real-time RT–PCR approaches were next adopted to evaluate DKK1 expression in normal tissues. Dickkopf-1 was found exclusively in the placenta (Figure 2A) as reported previously (Fedi *et al*, 1999). His restricted expression to the placenta was further confirmed in a second cDNA panel prepared from normal tissues (Figure 2B). We also exploited quantitative real-time RT–PCR to further validate this observation (Figure 2C). Critically, we confirmed DKK1 expression in the placenta with two distinct, commercially available mRNAs prepared from normal tissues (Origene, Rockville, MD, USA and BD-Clontech, Mountain View, CA, USA), and a weak detection in some normal tissues. Minimal DKK1 expression was further confirmed from normal breast tissues prepared from five different donors (Figure 2D).

### Preferential expression of DKK1 in hormone-resistant breast cancer, in familial cases and in primary tumours from patients with invaded axillary nodes

**Figure 3:**
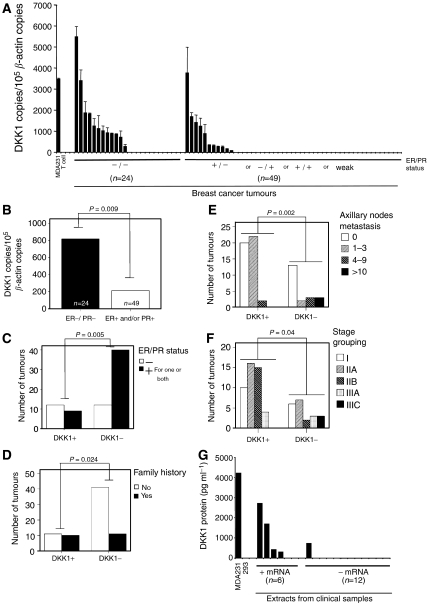
Dickkopf-1 expression from breast cancer clinical samples. (**A**) Complementary DNA was prepared from the indicated controls and clinical samples. Amplification was undertaken by real-time PCR and revealed by SYBR green staining. Amplification of the relevant amplicon was further confirmed by separation on agarose gel and ethidium bromide staining. (**B** and **C**) Samples from patients are clustered by ER and PR status, as evaluated by the local pathology clinical department (score: −: negative, +: positive, w: weakly positive, NA: not available). Dickkopf-1 levels from mRNA-positive tumours were clustered according to ER/PR status; statistical significance was evaluated by *t*-test (**B**). DKK1^+^ and DKK1^−^ samples were clustered according to ER/PR status; statistical significance was evaluated by the *χ*^2^ test (**C**). (**D**) DKK1^+^ and DKK1^−^ samples were clustered according to the reported familial history of breast cancer; statistical significance was evaluated by the *χ*^2^ test. (**E**) DKK1^+^ and DKK1^−^ samples were clustered according to the number of metastastic axillary nodes; statistical significance was evaluated by the *χ*^2^ test. (**F**) DKK1^+^ and DKK1^−^ samples were clustered according to the tumour stage grouping (AJCC); statistical significance was evaluated by the *χ*^2^ test. (**G**) A crude protein extract was prepared from available tumour samples as described in Materials and Methods. Dickkopf-1 secretion was determined by ELISA. The results presented are the average of at least two independent experiments.

To further characterise DKK1 expression in breast cancer tumours, we next assessed DKK1 expression profiles in clinical breast cancer specimens. Globally, DKK1 was detected in 21 out of 73 patient specimens evaluated (Figure 3A). The expression profile was clustered based on clinicopathological parameters characterising breast cancer. Dickkopf-1 expression profile was first analysed according to hormone receptors status (oestrogen and progesterone receptors; Figure 3A). Precisely, in DKK1^+^ tumours, the mRNA level was significantly higher in ER^−^/PR^−^ compared to other tumours (respectively 818 *vs* 213 DKK1 mRNA copies/10^5^ *β*-actin copies, *P*=0.009; Figure 3B). Also, as shown in Figure 3C, a statistical difference was demonstrated in the preferential DKK1 expression of hormone-independent tumours (*P*=0.005). This preferential expression was observed mainly in the absence of both hormone receptors. Specifically, only one out of the five ER^−^/PR^+^ tumours evaluated was DKK1^+^ (data not shown). Similar results were obtained with the ER^+^/PR^−^ group (one DKK1^+^ specimen of eight tested; data not shown).

We also evaluated expression depending on familial history, and 48% of DKK1^+^ tumours arose from women reporting familial cases of breast cancer (Figure 3D). When we compared the cohort tested, DKK1 expression was preferentially and significantly expressed in women with familial cases of breast cancer (*P*=0.024). Dickkopf-1 was also preferentially detected in primary tumours of patients with a higher number of metastatic axilliary lymph nodes, specifically with 10 or more invaded nodes (Figure 3E, *P*=0.002). Additionally, a higher proportion of DKK1 positive tumours was found in advanced breast cancer stages (American Joint Committee on Cancer, TNM stage grouping; Figure 3F). All tumours from patients with stage IIIC were DKK1^+^ (*P*=0.04), which includes tumours of any size presenting 10 or more metastatic axillary nodes. Finally, although it did not reach statistical significance, DKK1 expression was also documented in some of the most aggressive tumours, namely, in 39% of poorly differentiated histopathological grade 3 tumours and in 31% of tumours wider than 2 cm in their greatest diameter (data not presented). Furthermore, HER-2/*neu* overexpression was observed in only one DKK1-positive tumour. Dickkopf-1 expression was found in lobular as well as in ductal carcinomas and was not associated with recurrence. Altogether, cluster analysis revealed significant preferential DKK1 expression in familial and hormone-resistant breast cancers, which also encompassed the most aggressive tumours.

Importantly, DKK1 protein production was evaluated by ELISA in crude extracts prepared from breast cancer clinical specimens. As shown in Figure 3G, we detected DKK1 protein in four out of the six RT–PCR/DKK1^+^ samples. The two samples in which no DKK1 protein was detected had the lowest level of mRNA (<260 copies). Interestingly, when we evaluated 12 samples that were originally categorised as DKK1^−^ by RT–PCR, 11 were negative for the DKK1 protein and one was positive (the mRNA for this sample was prepared from an ER^−^/PR^−^ specimen).

Altogether, these data demonstrate DKK1 production (mRNA and protein) from breast cancer specimens, with a preferential expression pattern in tumours with poor outcomes.

### DKK1 is expressed in multiple tumour types

**Figure 4:**
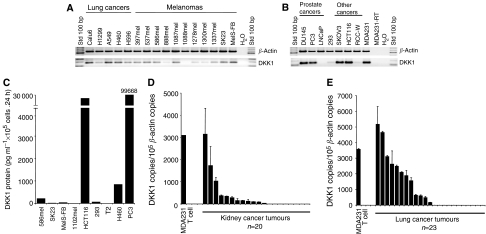
Dickkopf-1 expression in tumours derived from multiple sites. (**A** and **B**) Messenger RNA was prepared from the cancer cell lines indicated and RT–PCR analyses were performed with specific primers. Amplification was detected by ethidium bromide staining after electrophoresis migration in agarose gel. (**C**) The tumour cell lines indicated were plated for 24 h in 96-well plates as described in Materials and Methods. Supernatants were harvested, and DKK1 secretion was determined by ELISA. An average of at least two independent experiments is presented for each sample. (**D** and **E**) Complementary DNAs were prepared from the indicated controls and clinical samples of kidney (**D**) or lung (**E**) cancers. Amplification was undertaken by real-time PCR and revealed by SYBR green staining. Amplification of the relevant amplicon was further confirmed by separation on agarose gel and ethidium bromide staining. Legend: 293 are HEK-293 T HEK cells.

We next evaluated if DKK1 was expressed in tumours of other origins. Interestingly, as seen in Figure 4A and B, DKK1 expression was revealed in cell lines derived from lung cancer (five out of five), melanomas (nine out of 11), ovarian cancer (SKOV3) and colon cancer (HCT116). Dickkopf-1 was detected in two prostate cancer lines known to be hormone-independent (DU145 and PC3), but not in LNCaP, which is hormone-dependent (Figure 4B). The latter observation further corroborated previous findings on breast cancers, where DKK1 was preferentially expressed in hormone receptor-negative tumours (Figure 3C). Expression of DKK1 in some cancer cell lines was also confirmed by real time RT–PCR (Supplementary data, Figure 2). Dickkopf-1 protein secretion was evaluated in culture supernatants. As presented in Figure 4C, secretion was confirmed in cancer cell lines derived from the prostate (PC3), colon (HCT116), lung (H460) and one melanoma (586mel). Surprisingly, DKK1 was barely detected in two other melanoma lines that were positive for mRNA. We further evaluated DKK1 in cell extracts from these two melanoma lines, but no protein was detected, excluding the possibility that DKK1 is sequestered inside the cell (data not shown).

Finally, as depicted in Figure 4D, expression was evaluated in clinical samples prepared from kidney cancer, and we detected DKK1 at >200 copies/1 × 10^5^ copies of *β*-actin in six specimens (*n*=20). In addition, DKK1 was found in 11 out of 23 lung cancer specimens (Figure 4E). Altogether, these data suggest that DKK1 is a shared antigen expressed in multiple cancer types.

## DISCUSSION

Tumours have an aberrant protein expression profile as a consequence of genomic and proteomic alterations. Frequently, genes specialised in embryonic development are abnormally expressed in tumours. We describe here DKK1 expression in human tumours of various origins, including breasts, lungs and kidneys. Dickkopf-1, which is involved in some aspects of embryonic development, was detected in mature human tissues, mainly in the placenta, an observation reported by Fedi *et al* (1999). Interestingly, by analysing its expression profile in breast cancer patients, DKK1 appears in tumours with a poor outcome, specifically hormone-independent cases. Also, we reported preferential tumour expression in women with familial cases of the disease. Finally, we observed substantial DKK1 protein secretion in breast cancer lines, which was further confirmed in crude extracts prepared from breast cancer specimens.

In the embryo, DKK1 functions as a secreted protein interfering with the canonical Wnt pathway (Mao *et al*, 2002). In the absence of DKK1, Wnt interacts with two co-receptors, namely, LRP5/6 and Fz, which results in *β*-catenin accumulation and migration to the nucleus. Consequently, interaction with the transcription factor TCF delivers positive signals for cell proliferation (reviewed in Rothbacher and Lemaire, 2002; Brennan and Brown, 2004). Interestingly, in 1982, Nusse and Varmus (1982) identified the first *Wnt* gene as being a mammary oncogene, and several members of the Wnt family have been linked to cancer development, especially of the breast (reviewed in Li *et al*, 2000). Surprisingly, low levels of membranous *β*-catenin expression have been associated with significantly worse outcomes (Dolled-Filhart *et al*, 2006), which contradicts other studies (Lin *et al*, 2000; Chung *et al*, 2004). As such, there is still much debate about the link between tumour aggressiveness and *β*-catenin expression. Interestingly, DKK1 negatively affects the Wnt pathway. At the adequate time during embryogenesis, DKK1 is secreted and binds to the LRP5/6 co-receptor (Semenov *et al*, 2001), blocking interaction with secreted Wnt protein, causing *β*-catenin degradation and stopping TCF-regulated gene expression in the nucleus. This mechanism of DKK1 action is important in limb and head development (Glinka *et al*, 1998; Mukhopadhyay *et al*, 2001). Conversely, inhibition of the Wnt pathway by DKK1 initiates cardiogenesis early in vertebrate embryos (Marvin *et al*, 2001; Foley and Mercola, 2005).

Dickkopf-1 has been studied in the context of colon and gastric cancers. In colon cancer, Gonzalez-Sancho *et al* (2005) reported that the loss of DKK1 expression may open the door to cancer by removing the inhibitory effect on the Wnt/*β*-catenin pathway. Dickkopf-1 epigenetic inactivation may be a consequence of CpG methylation (Aguilera *et al*, 2006; Mikata *et al*, 2006). However, hypermethylation has been observed in only 17% of colon cancer clinical specimens, which indicates that this phenomenon is real but cannot be generalised (Aguilera *et al*, 2006). Also, the convincing mechanistic demonstration was performed mostly with cancer cell lines treated with the demethylating agent 5-aza-2′-deoxycytidine (DAC) and contradicts the fact that DKK1 is secreted by many highly proliferative cancer cell lines (Figures 1B, 3G and 4C) and is detected in many breast, kidney and lung cancer specimens.

Consequently, DKK1 may have either negative or positive consequences on development, depending on time and tissue distribution during embryogenesis. In cancer, DKK1 expression does not apparently alter cell growth, especially since we noted its expression in tumours with poor prognosis. The link between DKK1 and Wnt in the context of cancer progression is plausible and currently under investigation. Previous studies have shown that artificial DKK1 expression in some tumour lines with constitutive activation of the *β*-catenin pathways resulted in some decrease of cell viability but only in the presence of an oxidative stress inducer (Bafico *et al*, 2004). In cancer cell lines such as MDA231 and HCT116 where *β*-catenin is upregulated, the addition of inhibitors of the canonical Wnt pathway (other than DKK1) led to a marked reduction of free *β*-catenin (Bafico *et al*, 2004; Gregorieff and Clevers, 2005). However, according to our findings, these two cell lines already secrete high levels of DKK1 protein, which is known to be an inhibitor of the canonical Wnt pathway. Consequently, none of these *in vitro* studies correlate with the clinical observation we report here, about the presence of DKK1 protein in growing tumours from breast cancer patients. It is too early to speculate as to whether DKK1 plays a role in cancer similar to its known function in normal cells and in embryogenesis. It may be possible that DKK1 overexpression in *in vitro* systems may be masked by its other features when expressed at a physiological level. Still, a high cytoplasmic *β*-catenin level was found in patients with poor prognosis (Lin *et al*, 2000). DKK1 has been linked to other attributes specific to cancer cells. For example, Hall *et al* (2005) have recently reported that prostate cancer-derived DKK1 is involved in osteoblastic activity in bone metastases.

Dickkopf-1 could also be involved in particular phenotypes of hormone responsive tumours. We observed statistically significant preferential DKK1 expression in hormone receptor-negative (ER^−^/PR^−^) breast tumours (Figure 3B and C). Dickkopf-1 is regulated by progesterone in normal endometrial stroma cells (Tulac *et al*, 2006), but there is insufficient topical DKK1 expression in normal tissue for it to be linked to the expression profile reported here in breast cancer. Interestingly, Faivre and co-workers recently reported that the Wnt pathway can be upregulated by the progesterone receptor in breast cancer (Faivre and Lange, 2007). However, it is too soon to establish a link between DKK1 expression and the absence of hormone receptors. In fact, we observed DKK1 in two hormone-independent prostate cancer lines (DU45 and PC3; Figure 4B) but not in a hormone-dependent tumour (LNCaP). This expression profile is similar to that observed in breast cancer. Finally, we observed coexpression of DKK1 and HER-2/*neu* in breast cancer cells in only one out of the 21 DKK1^+^ tumours (data not shown). Consequently, only one of those tumours would be eligible for treatment with Herceptin^TM^, an antibody interfering with tumour progression. This further emphasises the necessity of finding additional targets for immunotherapy.

Interestingly, DKK1 could have potential applications as a secreted tumour marker for cancer diagnosis, staging and monitoring of relapse. Additional investigations are required to establish the feasibility of DKK1 protein detection in different body specimens or fluids.

In conclusion, as DKK1 is specifically expressed in common cancers, and absent from essential normal tissues, this protein is a potential TA for cancer immunotherapy. Its role as an inhibitor of the Wnt canonical pathway in normal cells aside, it may be possible to target DKK1 for a cytotoxic response through CD8^+^ T-cell recognition as a consequence of internal antigen processing leading to MHC class I presentation. In addition, a humoral response may be involved, as antigen-presenting cells can take up secreted tumour-derived DKK1 and elicit a CD4^+^ helper T-lymphocyte response. Importantly, considering that DKK1 is preferentially expressed in tumours from women with a family history, but absent from important normal tissues, the protein could be targeted in a preventive vaccine for women at risk of developing the condition. Actually, about 70–80% of women at high risk for breast cancer are predicted to develop the disease and, presently, with the exception of radical mastectomy, no effective prevention strategies are available.
