# Supplementary material for: The Wnt pathway regulator DKK1 is preferentially expressed in hormone-resistant breast tumours and in some common cancer types
Source: Br J Cancer. 2007 Jan 23;96(4):646–53. doi: 10.1038/sj.bjc.6603579 (PMC2360041; doi:10.1038/sj.bjc.6603579)
Supplement: Supplementary Data [file 6603579x1.doc]

**Forget *et al.***

**Supplementary data**

We highlighted genes overexpressed in breast cancers compared to libraries prepared from a wide array of normal tissues. β-actin and ubiquitin C genes were included as positive controls, and, as expected, the reported expression levels were relatively equivalent in the normal and tumor tissues selected. Also, we excluded several candidates found uniquely in libraries prepared from cultured cell lines (all genes identified by Hs, and EST 602281305F1; Table I).

**Table I. List of overexpressed candidate genes in breast cancer compared to normal tissues, identified by cDNA (EST) and the SAGE DGED from the CGAP server.** β-actin and ubiquitin C are referenced as ubiquitously-expressed genes.

Legend: ^1^ - Unigene ID and names when available; ^2^ - Number of positive sequences from libraries prepared from breast tumors or selected normal tissues, among all available tags or sequences. Values are reported as number of positives/1 X 10^6^ sequences; NA: not available; ^3^ - Numbers are bolded and underlined when p<0.05 and 5 times the value from normal cells; ^4^ - No assigned Unigene ID (EST prepared from osteocarcinoma). The expression level is subjectively reported from 0 to +++++.

**Forget *et al.***

**Supplementary data**

**Figure 1**

**Forget *et al.***

**Supplementary data**

**Figure 2**
